# Students’ perceptions of the rules and restrictions of gender at school: A psychometric evaluation of the Gender Climate Scale (GCS)

**DOI:** 10.3389/fpsyg.2023.1095255

**Published:** 2023-03-02

**Authors:** Jacqueline Ullman, Lucy Hobby, Natasha R. Magson, Hua Flora Zhong

**Affiliations:** ^1^Centre for Educational Research, School of Education, Western Sydney University, Penrith, NSW, Australia; ^2^Centre for Emotional Health, School of Psychological Sciences, Macquarie University, Sydney, NSW, Australia; ^3^Institute for Cultural Studies, Western Sydney University, Parramatta, NSW, Australia

**Keywords:** construct validity, factor analysis, school climate, gender, sexuality, education

## Abstract

Research in the field of gender and sexuality diversity and, more specifically, negative attitudes toward gender and sexuality diverse individuals, has acknowledged the relationship between individuals’ endorsement of sex-differentiated, normative gender roles and their attitudes toward gender and sexuality diversity. Such work has highlighted how normative expectations of gender, drawn from binarized gender roles, sit at the heart of homophobic and transphobic attitudes. Previous research in high school settings has measured gender and sexuality diverse (GSD) students’ experiences of homo/transphobic harassment as an element of ‘school climate’ with regard to acceptance of gender and sexuality diversity. However, to date, no research has measured GSD students’ perceptions about how valued binarized, gender-normative roles are at their schools, or the ways in which these norms might impact, and potentially constrain, these students’ academic and social schooling lives. The aim of the present study was to address this gap by developing and testing a new, multidimensional measure (the Gender Climate Scale; GCS) of GSD students’ ideas about how gender norms function within their school. Using a convenience sample of 2,376 Australian high school students who identify as GSD, the GCS was evaluated for its reliability, construct, and criterion validity and measurement invariance using confirmatory factor analysis (CFA) methods. Findings revealed that the estimates produced from the GCS were reliable, valid, and invariant across student reported gender (male/female/non-binary) and location (urban/rural). Criterion validity was supported, with GCS factors representing the promotion of traditional gender roles in the schooling environment negatively associated with perceived school belonging and inclusion and positively associated with bullying and social isolation. Future research with the GCS can inform school and curriculum policy on this important measure of school climate, not just for GSD students but for whole student cohorts.

## Introduction

Research shows that students who identify as same-sex attracted or who otherwise express their gender in ways that do not conform to social and performative norms associated with their sex are disproportionally victimized by their peers and report feeling less safe at school (Robinson et al., 2013; Lucassen et al., 2014). Homo/transphobic gendered harassment is largely understood as a manifestation of the larger socio-cultural phenomenon of gender socialization that occurs in schools, wherein boundaries are articulated for appropriate expressions of gender and the social marginalization of those who do not conform maintains a policed gender ‘order’ (Connell, 1996; Martino, 2000; Toomey et al., 2012). Further, this form of marginalization offers significant rewards for the perpetrators—namely, their ability to reinscribe their own “legitimate,” socially sanctioned masculinity or femininity (Kehily, 2002; Youdell, 2005; Meyer, 2008; Pascoe, 2013). As Ringrose and Renold (2010) write, within school-based cultures, “a range of ‘normative cruelties’ inhere in the social and cultural processes of becoming a recognizable gendered subject” (p. 575).

Homophobic name-calling is one way that students structure the boundaries of un/acceptable gender presentation and, while such language is used so pervasively so as to be almost ‘unseen’ by teachers (Ullman, 2021), students report using this language to identify other students’ perceived violation of gender norms (Slaatten and Gabrys, 2014). In particular, research has linked male students’ stronger hegemonic masculinity beliefs to their greater frequency of engaging in homophobic behaviors, including name-calling (Poteat et al., 2011). For adolescent girls, mediated performative standards of femininity and sexuality often function at the core of social cohesion, and their participation in “the activities that mark [ed] the limits of acceptable femininity” enables group bonding (Durham, 1999, p. 201). Social rewards and punishments can hinge on students’ outward performative expressions of gender; to wit, in a large-scale survey of Dutch teachers, when asked to classify the various forms of student-to-student bullying they had witnessed in the past year, nearly 75% of such incidences were classified as based on students’ appearance, with 37% based on gender expression (Collier et al., 2015).

Critically, educators are co-creators of the boundaries of normative gender ideologies, not only through the curriculum they deliver, but also through their quotidian reactions to gender-based harassment. In their study of 273 teachers in northern Italy, Zotti et al. (2019) found that teachers’ reported sexual prejudice, measured in part by teachers’ sense that gender nonconformity was a marker of same-sex attraction, was a significant and strong predictor of their tendency to legitimize students’ homophobic bullying as ‘normal’ or not that serious. Similarly, a 2015 survey of over 500 Dutch educators found that teachers with negative attitudes toward homosexuality were significantly less likely to report that they intervene during instances of student-perpetrated, gendered (homophobic) harassment (Collier et al., 2015). Of 1832 educators polled in the United Kingdom, 55% of secondary school teachers and 42% of primary school teachers reported that they do not always intervene when they hear this language; many perceive homophobic name-calling as harmless banter, which occurs too frequently within the school setting to intervene (Guasp et al., 2015). Accounts from young people echo these findings, with many GSD young people reporting that their teachers fail to intervene in instances of homophobic/transphobic harassment (Ullman, 2021) and, in some cases, function as the perpetrators of, and co-conspirators in, this victimization (Kosciw et al., 2018).

More generally, research has shown that teachers who view peer victimization at school as an ordinary element of growing up are less likely to punish acts of aggression, and more likely to respond with passivity (Troop-Gordon and Ladd, 2015). Further, in their research with US-based teachers, (Kochenderfer-Ladd and Pelletier, 2008) found that peer victimization was viewed as more gender-normative among boys than girls, and teachers who endorsed such views were least likely to intervene in instances of student victimization. Such in/actions co-create the boundaries for identities, expression, and behaviors that are seen as permissible and desirable, with implications for which harassment behaviors are notable and worthy of teacher intervention.

Undoubtedly, teachers’ and students’ attitudes about gender normativity—what is un/acceptable, what is ab/normal—play a significant role in school cultures. The everyday, informal, and institutionalized marginalization experienced by young people who express their gender in ways that do not conform to socially accepted/socially promoted “norms” is thus complex and involves multiple social actors. While these norms may be most keenly felt by GSD students, all students and other members of the school community are implicated.

### Research on school gender climate and its limitations

Using the term “school climate” to investigate and understand the schooling experiences of GSD youth has come to prominence, in no small part, by the ongoing work of the New York-based Gay, Lesbian and Straight Educational Network (GLSEN), whose first “National School Climate” survey for GSD students was run in 2001. Over the last two decades, their operationalization of school climate has included (1) frequency measures of school-based homo/transphobic victimization and (2) single-item measures of various school supports, including the presence of teachers who are supportive of gender and sexuality diversity, and students’ exposure to curriculum inclusive of gender and sexuality diversity (Kosciw et al., 2018).

Other large-scale quantitative research in this area has framed similar foci as an investigation of “school environment” for gender and sexuality diverse students, including (1) a uni-dimensional measure of same-sex attracted students’ accounts of how accepted lesbian, gay, and bisexual students are at their school and (2) same-sex attracted students’ personal experiences of homophobic victimization at school (Murdock and Bolch, 2005). While such explorations certainly add much to the field, given what is known about the relationship between negative attitudes toward gender and sexuality diversity and binarized conceptions of appropriate or acceptable gender expression as previously outlined, prior quantitative conceptions of school climate/environment as experienced by GSD youth seem to have overlooked this fundamental element, referred to here and elsewhere as “gender climate” (Ullman, 2018).

Qualitative research with young people has explored elements of gender climate through young people’s accounts of their personal experience of peer and teacher relationships, toward an understanding of the ways in which schools are complicit in the production of students’ gendered selves. Such studies have explored how normative, regulatory constructions of femininity and masculinity function in secondary school peer group cultures, particularly with regard to social in/exclusion (Martino, 2000; Renold, 2005; Youdell, 2005; Charles, 2010). Ringrose and Renold (2010), for example, have shown how normative gender roles at school shape differential views and reactions to harassment by students’ gender: socially sanctioned when actions align with gendered norms and viewed with aversion where perceived misalignment occurs. Youdell (2006) explores how students’ bodily activities, including physicality and adornments, reproduce, and are productive of, discourses of femininity and masculinity. Her work exposes teachers’ surveillance and its in/direct impact on students’ gender performativity and the boundaries of un/acceptable behaviors.

Qualitative explorations with GSD young people detail pressures to conform to hetero/cisnormative expectations and the ways in which both peers and educators act as enforcers of related boundaries (Ullman, 2014). While such studies frequently describe instances of school-based victimization and teachers’ responsiveness as elements of school climate or culture, they are more likely to account for nuance and complexity, inclusive of considerations related to gender (Smith et al., 2014). This work has certainly advanced the field in numerous ways, not least of which is exposing the multiple dimensions of schooling climate as related to gender and sexuality diversity. In many ways, it is not surprising that the field has not yet seen a quantitative scale measure which might be applied across schooling contexts which attempts to account for these dimensions. This is, no doubt, due to the difficulties in isolating and operationalizing the various elements which construct a gender climate in schools, including both teacher and peer influences, which “play a significant but not easily visible gate-keeping role in reproducing gender ideologies” (Stromquist, 2007).

### Theoretical basis and item development of the GCS

When GSD young people are provided with opportunities to share their schooling experiences with researchers, namely discussing school-based supports and stressors, several key elements are typically raised. The formal curriculum is experienced as a space of both validation or exclusion by GSD students, linked to students’ perceptions of their schools’ support for gender and sexuality diversity more generally (Kosciw et al., 2022). GSD students reflect on the importance of a feeling of freedom at school, both *freedom to* pursue a range of school activities/subjects, to express their diversity and to be ‘themselves’ and *freedom from* restrictive norms, frequently policed in the form of social rewards and punishments (Ullman, 2021). Research consistently highlights GSD students’ awareness of their teachers’ attitudes and expectations around gender expression, often communicated through direct instructions to GSD students to change elements of their behavior or appearance in order to ‘fit in’ to the school (Kosciw et al., 2022).

Alongside this substantial body of research, theorized factors and associated items for the GCS were informed by Ullman’s (2014) research including in-depth interviews with GSD young people, aged 16–19 from Sydney’s western suburbs. Findings highlighted how “gender climate” was constructed and maintained across three key locations at school: the organizational (institutional), the instructional, and the interpersonal. At the organizational level, students highlighted the restrictive nature of their required school uniforms, a standard feature of Australian schools, public or private, as well as school policies related to other elements of bodily appearance. Further, in keeping with Connell’s (2000) research exploring how school gender regimes manifest *via* the (gendered) meanings ascribed to particular academic subjects, GSD students in this research spoke how about the instructional ethos around various curriculum areas reinforced gender binaries and stigmatized students who participated in the “wrong’ classes or activities. Curricular silences in relation to “gender and sexuality were universally discussed by students, including students’ sense that their teachers did not value these topics and held negative, and potentially judgmental, feelings about GSD individuals. Lastly, at the interpersonal level, students spoke about social marginalization and victimization—perpetrated by peers and teachers alike—as directly related to, and seemingly excused by, their gender expression. GSD students sensed that the most valued and cared for students, by both their peers and their teachers, were those who were most conforming to cisnormative expectations. Thematic results from this research (Ullman, 2014) generated dimensions and item development in the production of the GCS.

### GSD students’ differential experiences across location and by gender

Research with GSD students shows differential experiences of gender-based harassment at school. Much variation has to do with state-level differences with regard to curriculum/policy visibility and teacher training (Ullman and Ferfolja, 2015), with GSD students living in rural locations in the United States significantly less likely to have received gender and sexuality diversity-inclusive education and more likely to report attending schools where teachers exhibit or appear to condone discriminatory practices (Kosciw et al., 2022). Further, in a national survey of Australian GSD high school students, students in regional and rural locations were significantly more likely than those in metropolitan locations to report having heard transphobic language and witnessing physical harassment of their GSD classmates within the last month at school, reporting less teacher intervention in these instances (Ullman, 2018).

A recent meta-analysis of gender stereotypes incorporating research with populations of children, adolescents, and young adults, highlights more restrictive prescriptive stereotypes for boys and men, inclusive of behaviors, interests, and gender expression (Koenig, 2018). Research with nearly 6,000 early high school students from the United States found that boys, but not girls, who perceived themselves as less gender typical, “experienced more loneliness and social anxiety in schools with more salient gender norms, even when accounting for both individual and school level victimization” (Smith et al., 2018, p. 1). For male students, attending schools with stricter gender norms predicted increased depressed mood, regardless of their personal gender normativity or atypicality (Smith et al., 2018). In GLSEN’s 2017 large-scale national survey of GSD students, more than half heard negative comments related to their peers’ masculinity either “often” or “frequently,” compared to two out of five students reporting the same about comments related to peers’ femininity (Kosciw et al., 2018).

### Associations between perceived gender climate and school wellbeing factors

Of the various measures of students’ wellbeing at school, school belonging has received a great deal of attention, due to its ability to predict multiple educational outcomes (Allen et al., 2021). High school students’ school belonging is among the strongest predictors of their educational engagement, positive attitudes toward learning (Ladd et al., 2009) and academic achievement (Fredricks et al., 2004; Juvonen, 2006). Importantly, research highlights the impact of supportive staff on this element of school wellbeing; where teachers are viewed by students as accepting (Hughes, 2011), respectful (Anderman, 2002), and caring (Roffey, 2012), students’ sense of school connection and belonging is enhanced.

Research with cohorts of GSD students has highlighted the impact of school gender climate on their various school-based wellbeing outcomes and, in particular, their school belonging. In terms of peer-based interactions, numerous large-scale research projects have linked GSD students’ self-reports of homophobic/transphobic (i.e., gender-focused) bullying to a lowered sense of school connection (Bradlow et al., 2017; Kosciw et al., 2018). Further research has modeled this relationship in a large cohort of trans/gender diverse high school students (*N* = 4,778), showing the significant, direct impact of peer victimization on students’ reported school belonging (Hatchel et al., 2019). Likewise, educators’ support of gender and sexuality diversity (or lack thereof) has been linked to GSD students’ sense of belonging at school, with homophobic/transphobic discrimination by teachers shown to be one of only two statistically significant predictors of this cohorts’ sense of belonging (Aerts et al., 2012). This relationship was echoed in a recent national survey of almost 23,000 GSD students in the United States, where students in schools with larger numbers of staff who were perceived to be supportive of GSD students experienced a greater sense of school belonging, performed better academically and had greater aspirations for tertiary study (Kosciw et al., 2022).

Further, research has shown that, where educators fail to appropriately respect trans/gender diverse students’ pronouns, these students are more likely to report trouble concentrating at school, report a drop in grades or leave school altogether (Jones et al., 2016). Similar relationships have been reported elsewhere, with school policy that impacts gender climate (e.g., provides protections for pronoun use/students’ choice of uniform) linked to trans/gender diverse students’ school belonging and retention (Kosciw et al., 2022). With respect to the Sydney-based qualitative research which informed item development for the current study (Ullman, 2014), variables associated with students’ experiences of school gender climate were clearly linked to their school-based wellbeing. For example, where young people reported a negative gender climate—where educators/peers did not respect or affirm gender and sexuality diversity and where traditional gender norms had a pervasive impact on social and academic freedoms—such experiences were implicated in students’ feelings of self-consciousness and isolation, with clear links to their ability to focus during class, their academic motivation and sense of school connection more broadly (Ullman, 2014).

Within the aforementioned literature, the reported correlations between factors related to school gender climate and varying elements of student wellbeing are typically between 0.10 to 0.29 and are considered indicative of low to moderate relationships (de Vaus, 2013), with a similar magnitude of associations expected in the present study. Specifically, factors related to teacher-student relations, including high expectations for success and teacher concern for students; student voice and agency; and school safety, including management of bullying and respect for diversity, were theoretically conceptualized as similar constructs to those represented in the new GCS measure and were thus utilized to assess the convergent validity of the GCS. Students’ sense of school connectedness, belonging and isolation, and experiences of bullying at school were theoretically proposed as outcomes of the schooling gender climate and these associations were proposed to measure the criterion validity of the GCS.

### Purpose of the current evaluation

While school climate for GSD students has often been conceptualized as focused on the frequency of homo/transphobic behaviors and teachers’ response, this represents only one central, and highly visible, dimension. Detail about what students learn in terms of gender and sexuality diverse identities and histories certainly adds another dimension, but these elements alone may not meaningfully capture the complex influence of gender normativity on these behaviors. The current project of operationalizing gender climate as a measurable feature of the high school environment addresses this gap in the literature. The development and psychometric evaluation of a self-report instrument designed to measure students’ personal perspectives on how gender normativity and diverse gender expression are treated, and potentially policed, at their schools, serves as an expansion of school climate research in the area of gender and sexuality diversity-inclusivity. In doing so, the authors aim to gain a more nuanced understanding of the factors which might contribute to gender-based, homo/transphobic harassment in school and thus contribute to this field of research.

## Materials and methods

### Participants and procedure

A total of 2,449 Australian youth who identified as gender and/or sexually diverse (GSD) completed an anonymous online survey. The data of 66 respondents were excluded as they failed to correctly answer one or more of the three attention check items placed throughout the survey and another seven were excluded due to malicious responses. The final dataset for analysis contained responses from a total of 2,376 participants. Respondents were aged between 13 and 18 years (*M_age_* = 15.6), and the majority were still attending secondary school (95.7%). Of the total sample, 1,394 identified as female (58.7%), 500 as male (21.0%), 211 as non-binary (8.9%), 169 reported being unsure of their gender (7.1%), and a further 102 described their gender in ‘another way’ (4.3%). In terms of sexuality, participants identified as: bisexual (34.6%), pansexual (17.8%), lesbian (15.8%), gay (11.2%), queer (7.0%), asexual (4.4%), unsure/questioning (5.1%), straight (0.6%), and other (3.5%). Participants were recruited through the Instagram and Facebook social media platforms using a small advertisement specifically targeting only youth who considered themselves to be gender and/or sexuality diverse. After clicking on the survey link provided, participants read the study information and consent form, and provided consent electronically by clicking “I agree.” To recruit additional participants, the information form also encouraged participants to share the study link with “other gender and sexuality diverse friends within Australia.” Taking approximately 35 min to complete, the anonymous survey was administered online *via* the Qualtrics platform with the items presented in a randomized order across participants. Participants who indicated that their age was younger than 13 or older than 18 were automatically screened out, as were those indicating that they lived outside of Australia.

### Instrumentation

A research report for this project outlines the full project scope and all included items (Ullman, 2021). The relevant included instrumentation for the psychometric evaluation of the Gender Climate Survey (GCS) are detailed below.

### Gender climate survey (GCS)

An initial pool of 73 items were generated to explore “gender-climate” as an element of the schooling experiences of GSD students. Scale development was informed by the literature in the field, as previously outlined, exploring the influence of gender norms as a facet of school climate, as experienced specifically by GSD students (Toomey et al., 2012; Slaatten and Gabrys, 2014; Kosciw et al., 2018). Items were developed in sets (i.e., hypothesized factors) to align with themes identified in qualitative (interview) research with GSD students conducted by the first author (Ullman, 2014), organized using the theoretical principals of stage-environmental fit theory (SEF; Eccles and Midgley, 1989; Eccles et al., 1993). Central themes related to the “gender-climate” of GSD students’ high schools included: (1) how supportive and respectful their school environment was of gender diversity and diverse gender expression; (2) the extent to which GSD students were enabled to express their gender through their appearance at school; (3) the extent to which the schooling environment reinforced traditional gender roles through discourse and school policy; (4) the extent to which gender and sexual diversity was incorporated into schools’ Health and Physical Education curriculum; (5) schools’ in/direct promotion of gender bias in subject selection; (6) the extent to which students’ grades/marks were perceived to be influenced by educators’ bias toward gender and sexuality diversity; (7) the extent to which GSD students perceived student popularity in their school to be based on adherence to traditional gender norms; and (8) support and acceptance of same-sex attraction and relationships. In total, 10 subscales were initially proposed, representing these eight general themes.

During the researchers’ iterative process of item development, wording was as faithful as possible to the framing of what Australian GSD young people experienced or observed, as outlined in the preliminary qualitative research (Ullman, 2014). Items were piloted with a small group of GSD young people (*n* = 4), recruited through snowball sampling techniques using the first author’s existing professional networks, using “think-aloud” piloting protocols (Ryan et al., 2012) to ensure that wording was clear, appropriate, and pitched at the correct reading level for this cohort.

To complete the survey, participants indicated how true they thought each of the 73 statements was using a 6-point Likert scale (1 = definitely false. 2 = mostly false, 3 = somewhat false, 4 = somewhat true, 5 = mostly true, 6 = definitely true). Although there is little consensus on the ideal number of response options to use in a Likert scale, six were chosen for the GCS as recent research has shown slight reductions in measurement precision when using five or less response options, and a lack of benefit for using more than six alternatives (Simms et al., 2019). Additionally, other research has shown that scales designed for children and adolescents are most reliable when offering less than seven response options (Borgers et al., 2004), with a label for each anchor point (Kline, 2005). In the current study, mean subscale scores were calculated with higher scores indicating greater endorsement of the truthfulness of each statement as applicable to their schooling environment.

### School belonging and isolation scale

The School Belonging and Isolation scale (SBI; Willms, 2003) items were replicated from the Program for International Student Assessment survey (PISA) which involves data collection from secondary students in over 90 countries worldwide. The six-item SBI scale measures adolescents’ perceived isolation (3 items; e.g., “I feel awkward and out of place”) and belonging (3 items; e.g., “Other students seem to like me”) in the schooling context. Participants rated their agreement with each statement on a 4-point Likert scale from 1 (strongly disagree) to 4 (strongly agree). Past research has shown both subscales to have good construct validity and reliability across gender and regional locations with adolescents aged 12–18 years old (Willms, 2003; Cueto et al., 2010; Magson et al., 2014). Cronbach alpha in the current study was satisfactory for both the belonging (*α* = 0.77) and isolation subscales (*α* = 0.80).

### Attitudes to school survey

The Attitudes to School Survey (ATSS; Department of Education and Training Victoria, 2018) was developed by the Victorian Education Department in Australia to measure students’ attitudes toward school. The current research used seven of the 21 ATSS factors deemed most relevant to the current research. These included: high expectations (6 items; e.g., “My teachers expect me to do my best”), teacher concern (4 items: e.g., “My teachers are interested in my well-being”), student voice (7 items; e.g., “I have the opportunity to participate in decision-making at the school”), management of bullying (3 items; e.g., “My school deals fairly with bullying problems”), respect for diversity (4 items; e.g., ‘Students in my school respect each other’s differences”), school connectedness (5 items, e.g., “I am happy to be at my school”), and bullying (5 items; e.g., “I have often been teased in an unpleasant way or called names at my school”).

Six of these factors were measured on a 5-point Likert scale from 1 (strongly disagree) to 5 (strongly agree) with the exception of the bullying subscale, which was measured dichotomously (0 = no, 1 = yes). Items across all scales were worded positively and mean scale scores were created for each participant following missing data replacement. Although the ATSS has not previously been validated, the Cronbach alpha values for all subscales in the present study were satisfactory (α = 0.77 to 0.92).

## Statistical analysis

Prior to the main analyses, all data were screened for normality and missingness using SPSS v27 (IBM Corp, 2017). Data were missing on all variables although the percentage of missing data was very low, ranging from 0.8 to 2%. Due to the low rate of missingness, the Expectation Maximization (EM) procedure was carried out in SPSS to replace missing values. All subsequent analyses were conducted on the EM-replaced data. Byrne (2010) and Hair et al. (2014) recommend that skewness values between −2 and + 2 and kurtosis values ranging from −7 to +7 can be considered normal for univariate distributions, and Brown (2015) and Kline (2015) recommend that for multivariate modeling, values of skewness that fall between −3 and + 3, and kurtosis in the range of −10 to +10 are acceptable, although cut-off values vary across the literature. In the current study, variable skewness values fell within the range of ±2 for all but one item (2.35) and kurtosis values ranged from 0.04 to 6.66. Due to the variable nature of cut-off scores across the literature, to account for the potential non-normality of some variables in the data, all Mplus analyses were conducted using the MLR estimator which is robust against non-normality (Li, 2016). As one-way ANOVAs have also been shown to be robust against non-normality, particularly with large sample sizes (>200; Blanca et al., 2017), conducting the nonparametric equivalent Kruskal–Wallis was not considered necessary. As there is some evidence to suggest that correlations can be affected by non-normality (Bishara and Hittner, 2015), both Spearman’s rho (nonparametric) and Pearson’s *r* (parametric) correlations were obtained during analysis. As the size and direction of the correlations were almost identical across methods, only the Pearson’s correlations are reported within this paper.

### Exploratory analysis of the factor structure

Prior to conducting the main analysis, the total sample was randomly split into two equal subsamples of 1,188 participants each using the random sample function in SPSS v27. Random sample 1 was used for the exploratory analyses (PCA and PFA), and random sample 2 was used for the confirmatory analyses (CFA) described below. PCA and PFA are the most widely used data reduction techniques in new instrument development. In the current study, the main aim was to reduce the number of items to produce the most parsimonious model possible while extracting enough components/factors to explain at least 60% of the variance. First, to identify the initial factor structure of the scale, all 73 items were entered into a PCA using Varimax rotation in SPSS v27 (IBM Corp, 2017). The PCA method uses all variances in the observed variables to extract the components and the orthogonal rotation (i.e., Varimax) assumes that the components extracted are uncorrelated (Tabachnick and Fidell, 2019). Component extraction criteria were based on eigenvalues greater than one and examination of the scree plot. Based on recommendations by Hair et al. (2014, 2017), criteria for the elimination of an item were: a communality value below 0.50, a factor loading below 0.50, and if an item loaded onto more than one factor at 0.40 or above. Second, as Tabachnick and Fidell (2019) state that a stable solution will be reproduced regardless of the extraction and rotation method employed, we reran the analysis using the Principal Axis Factoring (PFA) extraction method with Promax rotation. This method differs from PCA in that it accounts for potential measurement error by eliminating the unique variance in each of the observed variables and includes correlations between the factors extracted (Tabachnick and Fidell, 2019). Finally, to ensure that the EM procedure for dealing with missing data analysis did not bias the results, the PCA and PFA were rerun using listwise deletion on the data set containing missing values.

### Confirmatory factor analysis

Following the PCA and PFA, we ran a confirmatory factor analysis (CFA) with random sample 2 in Mplus version 8 (Muthén and Muthén, 1998/2017; Muthén and Muthén, 2015) to further validate the factor structure of the GCS measure. To evaluate how well the data fit to the hypothesized model we used the Root Mean Square Error of Approximation (RMSEA), the Comparative Fit Index (CFI), and the Standardized Root Mean Square Residual (SRMR). For the RMSEA, values below 0.050 represent an excellent fit and values up to 0.070-0.080 indicate good/acceptable fit, For the CFI, values greater than 0.95 are indicative of excellent fit, and values greater than 0.90 are indicative of good/acceptable model fit (Schumacker and Lomax, 1996). The SRMR ranges from 0 to 1 and values lower than 0.05 are indicative of a good-fitting model (Diamantopoulos and Siguaw, 2000), although it is important to note that some view these cut-off values as arbitrary (see Chen et al., 2008 for a discussion).

### Reliability analysis

Reliability analyses were performed for each of the proposed subscales of the GCS using Cronbach’s alpha. While there is no universal consensus regarding acceptable reliability values, a Cronbach’s alpha value of 0.70 or above is generally used as a point of reference (see Tabachnick and Fidell, 2019). Therefore, subscales with alpha values above 0.70 were deemed acceptable in the current study. Due to the growing body of literature highlighting the limitations and utility of using Cronbach’s alpha to determine the reliability of a measure (e.g., Revelle and Zinbarg, 2008; Cho and Kim, 2015; Sijtsma and van der Ark, 2015), we also computed the composite reliability for each subscale of the GCS using McDonald’s (1999) coefficient omega (ω).

### Invariance testing

To determine whether the theorized factor structure underpinning the GCS model was interpreted similarly across diverse groups, invariance testing was carried out across gender (male/female/non-binary) and location (urban/rural) groups. Specifically, three models in which parameters were held invariant in an increasingly stringent manner were conducted: Model one was the configural (baseline) model and was estimated freely across groups with no constraints; Model two was the metric model and held factor loadings invariant across groups; and Model three was the scalar model and held factor loadings and item intercepts invariant across groups (Byrne, 2004). To evaluate whether factorial invariance was achieved, changes in the goodness-of-fit indices between each successive model were compared to ensure that they did not exceed 0.01 for the CFI (ΔCFI) and.015 for the RMSEA (ΔRMSEA) as per the guidelines of Cheung and Rensvold (2002). A claim to strong factorial measurement invariance can be made if invariance across the three models is attained.

### Correlational analyses

To investigate the convergent and criterion validity of the new GCS measure, bivariate correlational analysis was used to investigate the associations between factors of the GCS and the ATSS and PISA subscales. The strength of the relationship coefficients was interpreted according to de Vaus’ (2013) guidelines of 0.01 to 0.09 (trivial), 0.10 to 0.29 (low to moderate), 0.30 to 0.49 (moderate to substantial), and 0.50 to 0.69 (substantial to very strong).

### Analyses of gender and location differences

To determine whether there were any significant differences between gender identity groups (male, female, non-binary, unsure, other) across the GCS factors, a one-way ANOVA with follow-up *post hoc* testing to determine the exact nature of any differences was conducted in SPSS. Furthermore, to determine any statistically significant differences between reported location groups (urban and rural) on the GCS factors, independent samples *t*-tests were carried out in SPSS. To adjust for making multiple pairwise comparisons in the one-way ANOVA and t-tests, we employed the method of Benjamini and Hochberg (1995) using a false discovery rate of 0.05 which resulted in a revised *value of p* of 0.019. Effect sizes were also calculated in the form of Cohen’s *d* for the one-way ANOVA, and Cohen’s *d* corrected for unequal sample sizes for the ANOVA *post hoc* and t-test pairwise comparisons. Cohen’s *d* effect sizes were interpreted as: 0.2 small, 0.5 medium, and 0.8 large.

## Results

### Exploratory factor analyses

The initial principal component analysis (PCA) on the 73 proposed items produced a 10-factor solution explaining 66.10% of the total variance, although an examination of the scree plot and the variance explained suggested that the last three of the 10 extracted factors accounted for very little of the variance. Five items produced a low communality (< 0.5), an additional three items failed to sufficiently load into any of the components extracted (< 0.5), and a further 12 items cross-loaded across components resulting in the removal of 20 items in total. A second PCA was run on the remaining 53 items which extracted seven components with eigenvalues greater than one and explained 65.55% of the total variance. Four items had a low communality, four items had low factor loadings, and an additional three items cross-loaded resulting in the deletion of a further 11 items. The third PCA with the remaining 42 items resulted in a seven-factor solution explaining 70.14% of the total variance. All communalities and factor loadings were 0.5 or above, and all items loaded onto one factor only.

To test the stability of the solution, we reran the model using the PFA extraction method with Promax rotation. The results replicated the PCA solution explaining 61.74% of the variance with the same items uniquely loading onto the same seven factors, with only minor variations in the magnitude of the factor loadings and communalities (all > 0.5; see the PCA and PFA rotated matrices in Supplementary Material Appendix A). Finally, rerunning the PCA and PFA analysis in the dataset containing the missing values using listwise deletion did not substantively change the results of either analysis in any way. Based on the examination of item groupings, the seven extracted factors were subsequently named: 1. School’s Acceptance and Support of Gender and Sexuality Diversity (13 items; SAS-GSD); 2. Reinforcement of Traditional Gender Difference (3 items; RTGD); 3. Freedom of Subject Selection (3 items; FSS); 4. Freedom of Appearance Expression (3 items; FAE); 5. Inclusive Health and Physical Education Curriculum (4 items; IC); 6. Academic Fairness (5-items; AF); and 7. Popularity Based on Gender Norms (11 items; PBGN).

### Confirmatory factor analysis

**Figure 1:**
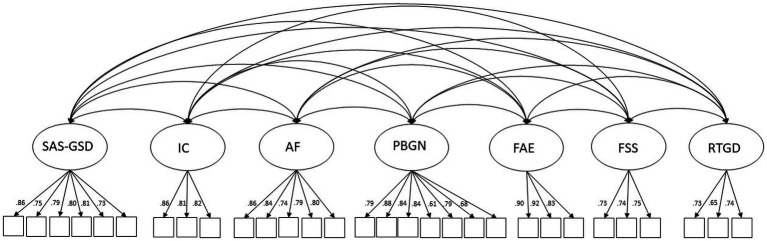
Final Gender Climate Scale (GCS) Model.

The 42-item 7-factor CFA model using random sample two produced an adequate fit to the data (*x^2^* = 3585.58, *df* = 798, CFI = 0.91, RSMEA = 0.052, SRMR = 0.038). All 42 items loaded positively and significantly onto their designated factors (*p* < 0.001) with acceptable loadings ranging from 0.65 to 0.92. Correlations between the factors were all significant (*p* < 0.001) and indicated that although related, each factor was a distinct construct with correlations between factors ranging from −0.18 to 0.58. Examination of the modification indices and standardized residuals for covariances indicated that the removal of several items would improve the model fit. Item pairs with the largest modification indices were considered for removal first. Before removal, the standardized residuals (z-scores) for covariances among items were examined for significance (*z*-scores >1.96) and the item with the largest residuals with multiple items was removed. Items were removed one at a time resulting in the removal of 12 items in total (7 items from SAS-GSD, 4 items from PBGN, and 1 item from ICC), all of which had very high modification indices and significant residual covariances (*p* < 0.001) with multiple items. The final result was a 30-item seven-factor model reflecting the factors described above. The model provided an excellent fit to the data (*x^2^* = 914.45, *df* = 384, CFI = 0.99, RSMEA = 0.034, SRMR = 0.034). All factor loadings were positive and significant, ranging from 0.60 to 0.92, and the correlations between factors ranged from −0.19 and 0.59. The final GCS model is depicted visually in Figure 1 and the items are listed in Supplementary Material Appendix B.

### Final model descriptive statistics

The mean scale scores for each of the final factors for the total sample, and stratified by self-identified gender and location, can be found in Table 1. As shown, AF was most highly endorsed by the total sample and all subsamples. In contrast, the IC factor had the lowest means across all participant categories. Correlations between the GCS factors are displayed in Table 2. All correlations were significant (*p* < 0.001) and ranged in size from small (*r* = −0.15) to large (*r* = 0.55). The relations between factors were all in the theoretically anticipated direction, with reinforcement of traditional gender roles, and popularity based on those gender roles, being negatively associated with freedom of subject selection and appearance expression, academic fairness, inclusive curriculum, and school acceptance and support of students’ gender and sexual diversity.

**Table 1:** Factor means and standard deviations for the total sample and by self-defined gender and location.

Factor	Mean (SD)								Gender						Location		Total (*N* = 2,376)	Male (*n* = 500)	Female (*n* = 1,394)	Non-binary (*n* = 211)	Unsure (*n* = 169)	Other (*n* = 102)	Urban (*n* = 1,301)	Rural (*n* = 806)
RTGD	4.17 (1.12)	4.13 (1.14)	4.15 (1.11)	4.24 (1.23)	4.30 (1.06)	4.33 (1.10)	4.11 (1.14)	4.24 (1.12)
FSS	3.98 (1.37)	3.81 (1.38)	4.10 (1.34)	3.77 (1.45)	3.75 (1.46)	3.99 (1.33)	4.04 (1.37)	3.82 (1.37)
FAE	3.12 (1.83)	2.85 (1.78)	3.24 (1.86)	3.08 (1.79)	3.20 (1.81)	2.82 (1.76)	3.13 (1.87)	3.05 (1.79)
IC	2.03 (1.42)	2.04 (1.40)	2.06 (1.45)	2.01 (1.34)	1.94 (1.43)	1.81 (1.25)	2.09 (1.46)	1.93 (1.34)
AF	5.32 (0.88)	5.32 (0.89)	5.40 (0.81)	5.10 (1.04)	5.19 (0.92)	5.08 (1.17)	5.35 (0.87)	5.26 (0.92)
PBGN	4.71 (1.12)	4.59 (1.20)	4.71 (1.10)	4.87 (1.07)	4.81 (1.11)	4.86 (1.07)	4.70 (1.14)	4.73 (1.08)
SAS-GSD	3.17 (1.38)	3.05 (1.40)	3.28 (1.37)	3.06 (1.38)	2.94 (1.35)	2.75 (1.36)	3.24 (1.39)	3.06 (1.35)

RTGD, Reinforcement of Traditional Gender Difference; FSS, Freedom of Subject Selection; FAE, Freedom of Appearance Expression; IC, Inclusive Health and Physical Education Curriculum; AF, Academic fairness; PBGN, Popularity Based on Gender Norms; and SAS-GSD, School’s Acceptance and Support of Gender and Sexuality Diversity.

**Table 2:** Bivariate zero-order correlations between the seven factors of the gender climate survey.

	RTGD	FASS	FAE	IC	AF	PBGN	ASGSD	*α*/*ω*
RTGD	1							0.75/0.75
FSS	−0.32*	1						0.79/0.79
FAE	−0.25*	0.29*	1					0.92/0.92
IC	−0.15*	0.18*	0.25*	1				0.88/0.88
AF	−0.25*	0.39*	0.30*	0.22*	1			0.90/0.90
PBGN	0.37*	−0.34*	−0.26*	−0.17*	0.21*	1		0.91/0.91
SAS-GSD	−0.38*	0.48*	0.55*	0.34*	0.48*	−0.46*	1	0.91/0.91

RTGD, Reinforcement of Traditional Gender Difference; FSS, Freedom of Subject Selection; FAE, Freedom of Appearance Expression; IC, Inclusive Health and Physical Education Curriculum; AF, Academic fairness; PBGN, Popularity Based on Gender Norms; SAS-GSD, School’s Acceptance and Support of Gender and Sexuality Diversity; α, Cronbach’s alpha; and ω, coefficient omega.

* Correlation significant at the 0.001 level (2-tailed).

### Reliability analyses

As shown in Table 2, the 30-item seven-factor GCS demonstrated good internal consistency for all individual subscales and the total scale (*α*’s = 0.75–0.92). Examination of the inter-item correlation matrix indicated that items within each subscale were well correlated, and item-total statistics indicated that deletion of any of the included items would not result in further improvement. Also shown in Table 3, computation of coefficient omega revealed that all values were acceptable in the current study (*ω*’s = 0.75–0.92).

**Table 3:** Measurement invariance of the Gender Climate Scale.

Model	*χ2*	*df*	CFI	TLI	RMSEA [90% CI]	Comp Model	∆CFI	∆TLI	∆RMSEA	Decision
Gender (*N =* 1,188; male *n* = 254; female *n* = 708; non-binary *n* = 226)										
M1: Configural	1783.04	1,152	0.966	0.961	0.037 [0.034, 0.041]					
M2: Metric	1810.13	1,198	0.967	0.964	0.036 [0.033, 0.039]	M1	+0.001	+0.003	−0.001	Accept
M3: Scalar	1916.25	1,244	0.964	0.963	0.037 [0.037, 0.040]	M2	−0.003	−0.001	+0.001	Accept
Location (*N* = 1,049; urban *n* = 627; rural *n* = 422)										
M1: Configural	1301.39	768	0.966	0.962	0.036 [0.033, 0.040]					
M2: Metric	1321.24	791	0.966	0.963	0.036 [0.032, 0.039]	M1	0.000	+0.001	0.000	Accept
M3: Scalar	1344.12	814	0.966	0.964	0.035 [0.032, 0.039]	M2	0.000	+0.001	−0.001	Accept

CFI, Comparative Fit Index; TLI, Tucker Lewis Index; RMSEA, Root Mean Square Error of Approximation; and, Comp Model, Comparison Model.

### Invariance analyses

Table 3 presents the findings from the multi-group invariance analyses. For gender (male/female/non-binary), Model one (baseline) fit the data well and the subsequent change in goodness-of-fit indices to Model two (factor loadings invariant) was negligible (ΔCFI = 0.001; ΔTLI = −0.003; ΔRMSEA = 0.000). In comparing Model two to Model three (factor loadings and intercepts), the change in model fit indices did not exceed the levels required for invariance ΔCFI = −0.006; ΔTLI = +0.002; ΔRMSEA = −0.001), supporting scalar invariance of the GCS across gender.

Across location groups (urban/rural), Model one demonstrated a good fit with the data. There were no changes in CFI and RMSEA fit indices from Model one to Model two when factor loadings were held invariant and only minimal increase in the TLI (ΔTLI = +0.001). Thus, metric invariance was supported. When both factor loadings and intercepts were held invariant from Model two to Model three, the changes in fit indices were well below the recommended guidelines (ΔCFI = 0.000; ΔTLI = +0.001; ΔRMSEA = −0.001). Hence, scalar invariance of the GCS across localities was supported.

### Convergent and criterion validity

Pearson’s correlations were used to assess the convergent and criterion validity of nuanced associations between the factors of the GCS and the ATSS and PISA constructs. As presented in Table 4, all associations between factors were significant (*p* < 0.001), although they varied in magnitude from low to very strong (de Vaus, 2013). Broadly, all associations were in the expected direction, with factors representing the promotion of traditional gender roles in the schooling environment (i.e., RGTD and PBGN) negatively associated with factors which measured positive aspects of the school climate, and conversely, factors of the GCS representing gender-affirming schooling practices (i.e., FSS, FAE, IC, AF, and SAS-GSD) positively associated with adaptive school climate constructs measured by the ATSS and PISA scales.

**Table 4:** Bivariate correlations between factors of the ATSS/PISA and the gender climate survey.

Gender climate survey factors
ATSS factors	RTGD	FSS	FAE	IC	AF	PBGN	SAS-GSD
High expectations	−0.21*	0.26*	0.11*	0.14*	0.38*	−0.18*	0.28*
Teacher concern	−0.22*	0.25*	0.17*	0.20*	0.33*	−0.25*	0.36*
Student voice	−0.28*	0.35*	0.26*	0.25*	0.37*	−0.32*	0.43*
Management of bullying	−0.27*	0.39*	0.25*	0.25*	0.42*	−0.31*	0.51*
Diversity	−0.31*	0.43*	0.27*	0.26*	0.43*	−0.38*	0.58*
School connectedness	−0.27*	0.33*	0.21*	0.21*	0.35*	−0.32*	0.47*
Bullying	0.28*	−0.30*	−0.15*	−0.12*	−0.29*	0.26*	−0.36*
School belonging and isolation subscales (PISA)							
Belonging	−0.19*	0.27*	0.13*	0.18*	0.28*	−0.29*	0.35*
Isolation	0.20*	−0.26*	−0.16*	−0.14*	−0.26*	0.31*	−0.35*

ATSS, Attitudes Toward School Survey; RTGD, Reinforcement of Traditional Gender Difference; FSS, Freedom of Subject Selection; FAE, Freedom of Appearance Expression; IC, Inclusive Health and Physical Education Curriculum; AF, Academic fairness; PBGN, Popularity Based on Gender Norms (PBGN); SAS-GSD, School’s Acceptance and Support of Gender and Sexuality Diversity; and, PISA, Program for International Student Assessment. *Correlation significant at the 0.001 level (2-tailed).

More specifically, in assessing convergent validity, subscales measuring similar content were more closely associated. For example, the GCS RTGD (*r* = −0.31), FSS (*r* = 0.43), AF (*r* = 0.43), PBGN (*r* = −0.38), and SAS-GSD (*r* = 0.58) factors were most strongly associated with the ATSS Diversity factor; the GCS AF factor held the largest association with the ATSS high expectations factor; and the GCS FAE and IC factors held the smallest magnitude relationships with ATSS high expectations and teacher concern. In examining criterion validity, varying strengths of associations were found, with the GCS SAS-GSC factor moderately to substantially related to the ATSS factors of students’ sense of school connectedness (*r* = 0.47) and experiences of bullying (*r* = −0.36) and the PISA school belonging (*r* = 0.35) and isolation (*r* = −0.35) subscales.

### Gender and location differences in the perceived gender climate of the school environment

A one-way ANOVA was used to investigate gender mean differences on each of the GCS factors. The assumption for homogeneity of variance was met for all factors (*p* > 0.05) except for the AF factor, *F* (4, 2,371) = 1.95, *p* < 0.001, thus the Games-Howell statistic was when interpreting significant mean differences on this factor. The results revealed significant gender differences on five of the seven GCS factors with small to negligible effect sizes: SAS-GSD [*F* (4, 2,371) = 7.09, *p* < 0.001, *d* = 0.22], FSS [*F* (4, 2,371) = 6.89, *p* < 0.001, *d* = 0.21], AF [*F* (4, 2,371) = 8.80, *p* < 0.001, *d* = 0.25], PBGN [*F* (4, 2,371) = 4.13, *p* = 0.011, *d* = 0.16], and FAE [*F* (4, 2,371) = 5.02, *p* < 0.001, *d* = 0.18]. For the SAS-GSD factor, females scored significantly higher those describing their gender as ‘other’ (*p* = 0.002, *d* = 0.39). On the FSS factor, significant differences were again found for females who reported significantly more freedom of subject selection than males (*p* < 0.001, *d* = 0.21), non-binary (*p* = 0.011, *d* = 0.24), and those unsure of their gender (*p* = 0.019, *d* = 0.08). For the AF factor, females perceived significantly higher levels of academic fairness when compared to those who identified as non-binary (*p* = 0.001, *d* = 0.36). For PGBN, although males scored higher than non-binary students (*p* = 0.026), this was no longer significant when accounting for the adjusted *value of p* (>0.019). For FAE, females scored significantly higher than males (*p* < 0.001, *d* = 0.23).

Independent samples *t-*tests revealed significant differences between location groups on the GCS SAS-GSD, RTDGF, FAE, and IC factors (*p* < 0.019). Specifically, young people residing in urban locations scored significantly higher than young people residing in rural locations on the SAS-GSD [*t* (2105) = 2.72, *p* = 0.007, *d* = 1.38], FSS [*t* (2105) = 3.49 *p* < 0.001, *d* = 1.37], and IC [*t* (2105) = 2.44 *p* = 0.015, *d* = 1.42] factors. Lastly, those from urban locations reported significantly lower on the RTGD factor than those from rural locations [*t* (2105) = −2.56 *p* = 0.011, *d* = 1.13].

## Discussion and conclusion

The present study aimed to perform a psychometric evaluation of the scores produced from the multidimensional GCS instrument through the generation of discrete factors and an assessment of their measurement invariance and criterion validity. The initial exploratory analyses using PCA and PFA resulted in the 10 factors initially conceptualized being refined to seven. None of the items originally hypothesized to sit as two factors related to (1) open classroom conversation about gender and sexuality diversity and (2) teachers’ affirmation of gender diversity were retained. These items referenced classroom teachers’ direct, vocal endorsement of gender and sexuality diversity, arguably the most challenging area to shift—particularly within the Australian context, where recent moral panics have positioned such topics as dangerous for educators’ engagement (Ullman, 2022). Further, three items initially proposed on each of two unique factors related to (1) acceptance of non-conforming gender expression and (2) acceptance of same-sex attraction in the school environment were retained to form a single factor, subsequently renamed “school's acceptance and support of gender and sexual diversity” (SAS-GSD). The manner in which gender diverse and sexuality diverse individuals tend to be considered in concert as a singular minority cohort within schools perhaps offers some explanation for this outcome.

The other six factors were retained in their original structure. Good internal consistency was demonstrated for estimates emanating from each of the seven factors of the GCS, as well as for the total scale measure. The scores derived from the measure were shown to be invariant across participants’ gender and location and criterion validity was established through an examination of bivariate correlations with known measures in use with this population of school-aged adolescents, with all associations in the expected direction.

Through our psychometric evaluation of this original measure, several notable findings were observed. Firstly, of the various dimensions of gender climate, GSD students were least likely to endorse factors associated with formalized school policies or curricular approaches present within their school environment. Specifically, students’ mean scores were the lowest for an inclusive HPE curriculum (IC) and freedom to express their appearance (FAE) with respect to uniform policies, rules surrounding hair length/style, etc. Findings are in keeping with research from the Australian context which highlights the contextual and cultural challenges which arise from near-universal uniform mandates (Jones et al., 2016; Ferfolja and Ullman, 2020) and underscore the infrequency of gender and sexuality diversity-inclusive curriculum in HPE (Fisher et al., 2019; Ezer et al., 2020). Encouragingly, the young people surveyed as part of this research were most likely to agree that their teachers were unbiased in their assessment of students’ achievement (AF).

Sub-cohort analyses of mean score differences across the seven factors highlighted that, as compared to students living in rural areas, students living in major city/urban areas reported schooling environments which were significantly more accepting and supportive of gender diversity and same-sex attraction (SAS-GSD); offered significantly more freedom of subject selection (FSS); and offered a HPE curriculum significantly more inclusive of gender and sexuality diversity (IC). Furthermore, GSD students in urban areas were significantly less likely to perceive that traditional gender roles were maintained through teacher discourse with students within the school (RTGD). These findings are in keeping with both national (Ullman, 2018) and international (Kosciw et al., 2022) research which has underscored the impact of geographical differences in school culture, curriculum policy, and teaching practices that serve to engender even stricter boundaries for gender normativity within rural locations.

Furthermore, female-identifying students reported significantly more freedom of subject selection at school (FSS) than most other gender cohorts. Likewise, female students were significantly more likely to endorse their teachers’ academic fairness (AF) and their school’s general acceptance of same-sex relationships and social transition for trans/gender diverse students (SAS-GSD). Participants with a non-binary gender identity, inclusive of students who were unsure of their gender and students who identified their gender as other than male/female, scored lower as a group on several GCS factors, including an inclusive HPE curriculum (IC); teachers’ academic fairness (AF); freedom of subject selection (FSS); and general school acceptance and support of gender and sexuality diversity (SAS-GSD). These results echo other research in the area which articulates the impact of a schooling system characterized by binary notions of gender (Paechter et al., 2021), particularly for non-binary young people (Hill et al., 2021; Travers et al., 2022).

Unsurprisingly, gender climate, as measured by the GCS, was associated with various measures of school-based wellbeing for this cohort of students, with all associations in the expected directions. For this cohort of students, where their teachers were viewed as promoting an inclusive gender climate through acceptance, support, and academic fairness, they were more likely to feel connected to school and have the sense that their schools valued them, and diversity, more generally. While results from cross-sectional research should be interpreted with some caution, it is encouraging to see that hypothesized relationships were present between factors of the GCS and measures of school-based wellbeing, both those conceptualized as pointing to convergent validity and those positioned as measures of related outcomes of interest. In particular, given the importance of students’ sense of belonging and connection to the school environment (Allen et al., 2021) and previous research with GSD students which has shown this to be impacted by elements of students’ experienced/perceived school climate (Hatchel et al., 2019; Kosciw et al., 2022), the magnitude of association between the SAS-GSD factor as an element of gender climate and students’ reported belonging/isolation as measured by the PISA scales stands out as affirming evidence of the relevance of this instrument. While not all reported correlations between factors of the GCS and factors of the ATSS/PISA measures exceeded a “moderate” ranking in strength (de Vaus, 2013), this is likely related to the fact that these measures were written to be domain-general rather than specific; for instance, were the ATSS “high expectations” factor to be measured as specific to teachers’ high expectations for GSD students, the strength of associations with the GCS subscales would undoubtedly increase.

Taken together, the findings from the present research are suggestive of students’ discernment of limiting gender normative schooling cultures that pervade the macro schooling curriculum, structure, and ethos. Importantly, these appear as distinct from the individual perspectives and practices of teachers who are operating within this wider “culture of limitation” (Ferfolja and Ullman, 2020), at least in regard to student academic performance. Furthermore, teachers’ capacity to separate gender from their evaluations of student achievement appears to be the very minimum requirement for fostering students’ sense of connectedness and belonging at school. Indeed, research (Ullman, 2022) which has isolated the experiences of trans/gender diverse young people from the sample on which the present paper is based, found that students’ sense of their teachers’ interpersonal valuing of them both academically and socially (high expectations for success and teacher concern) explained additional variance in school belonging above demographic and school contextual factors. A positive school climate that is accepting and supportive of gender and sexuality diversity added an even further unique contribution to students’ sense of belonging at school.

The GCS measure, developed based on the lived experiences of GSD students, and with GSD young people involved in item/construct co-review, offers the field its first validated measure of this construct. The rigorous and state-of-the-art psychometric evaluation presented in this paper demonstrates that this instrumentation is fit for purpose for use with cohorts of high school students. Notwithstanding the many strengths of the present research, there are some limitations to acknowledge. The research within this paper was based on responses drawn from a non-probability sample, using a cross-section of GSD young people, which cannot be considered representative of all GSD young people. Furthermore, the psychometric soundness of the measure cannot be assured for young people from more varied settings or communities, for example students who identify as heterosexual and cisgender or are from culturally or linguistically diverse backgrounds, as these young people were not explicitly considered in the development of the GCS. Furthermore, while the ATSS factors were used as one of the representative measures to assess the validity of the GCS, the ATSS was written to assess students’ general perceptions of their school-based wellbeing; the ATSS is not itself a measure of gender climate, as no such measure exists. Hence, the results should be considered with these limitations in mind.

Findings from this research have practical implications for educators, beyond the substantive results reported herein. Firstly, this research highlights the need for teacher professional development that identifies gender climate as an element of broader school culture and identifies the impact of cisnormativity and transphobia in the classroom (De Pedro et al., 2016; Fenaughty et al., 2019); this appears to be particularly critical for educators teaching in rural areas. Furthermore, this validated measure can aid teachers’ understanding of how gender climate manifests and is experienced by GSD-identifying students. Future research which validates this measure in a representative cohort of heterosexual and cisgender-identifying students would allow for important comparisons across these groups.

## Data availability statement

The datasets presented in this article are not readily available because additional analyses are currently in progress using this material. Requests to access the datasets should be directed to j.ullman@westernsydney.edu.au

## Ethics statement

The studies involving human participants were reviewed and approved by Western Sydney University Human Research Ethics Committee. Written informed consent from the participants’ legal guardian/next of kin was not required to participate in this study in accordance with the national legislation and the institutional requirements.

## Author contributions

JU conceptualized and executed the study. HZ undertook foundational analyses of a previous iteration of the GCS, used to guide the development of the final instrumentation presented here. LH and NM analyzed the data. JU, LH, and NM drafted the manuscript, and reviewed and edited the manuscript. All authors read and agreed to the published version of the manuscript.

## Funding

This project was supported by funding from Western Sydney University through their Researcher Development Scheme Award.

## Conflict of interest

The authors declare that the research was conducted in the absence of any commercial or financial relationships that could be construed as a potential conflict of interest.

## Publisher’s note

All claims expressed in this article are solely those of the authors and do not necessarily represent those of their affiliated organizations, or those of the publisher, the editors and the reviewers. Any product that may be evaluated in this article, or claim that may be made by its manufacturer, is not guaranteed or endorsed by the publisher.

## Supplementary material

The Supplementary material for this article can be found online at: https://www.frontiersin.org/articles/10.3389/fpsyg.2023.1095255/full#supplementary-material.
