# Supplementary material for: Students’ perceptions of the rules and restrictions of gender at school: A psychometric evaluation of the Gender Climate Scale (GCS)
Source: Front Psychol. 2023 Mar 2;14:1095255. doi: 10.3389/fpsyg.2023.1095255 (PMC10019353; doi:10.3389/fpsyg.2023.1095255)
Supplement: Supplementary file 1 [file Data_Sheet_1.docx]

Appendix A – Component and Pattern Matrices for the Final 30-item PAF and PCA solution

|  | Principal Axis Factoring Results | | | | | | | Principal Component Analysis | | | | | | |
| --- | --- | --- | --- | --- | --- | --- | --- | --- | --- | --- | --- | --- | --- | --- |
|  | Pattern Matrix | | | | | | | Rotated Component Matrix | | | | | | |
| Variable | F1 | F2 | F3 | F4 | F5 | F6 | F7 | F1 | F2 | F3 | F4 | F5 | F6 | F7 |
| ASGSD_1 | .89 |  |  |  |  |  |  | .80 |  |  |  |  |  |  |
| **ASGSD_2** | .87 |  |  |  |  |  |  | .79 |  |  |  |  |  |  |
| **ASGSD_3** | .85 |  |  |  |  |  |  | .76 |  |  |  |  |  |  |
| ASGSD_4 | .81 |  |  |  |  |  |  | .76 |  |  |  |  |  |  |
| ASGSD_5 | .80 |  |  |  |  |  |  | .75 |  |  |  |  |  |  |
| **ASGSD_6** | .80 |  |  |  |  |  |  | .75 |  |  |  |  |  |  |
| **ASGSD_7** | .80 |  |  |  |  |  |  | .74 |  |  |  |  |  |  |
| ASGSD_8 | .77 |  |  |  |  |  |  | .74 |  |  |  |  |  |  |
| ASGSD_9 | .77 |  |  |  |  |  |  | .73 |  |  |  |  |  |  |
| **ASGSD_1** | .74 |  |  |  |  |  |  | .73 |  |  |  |  |  |  |
| ASGSD_1 | .73 |  |  |  |  |  |  | .71 |  |  |  |  |  |  |
| **ASGSD_1** | .68 |  |  |  |  |  |  | .69 |  |  |  |  |  |  |
| ASGSD_1 | .63 |  |  |  |  |  |  | .64 |  |  |  |  |  |  |
| **PBGN_1** |  | .85 |  |  |  |  |  |  | .81 |  |  |  |  |  |
| **PBGN_2** |  | .82 |  |  |  |  |  |  | .80 |  |  |  |  |  |
| **PBGN_3** |  | .80 |  |  |  |  |  |  | .79 |  |  |  |  |  |
| PBGN_4 |  | .80 |  |  |  |  |  |  | .78 |  |  |  |  |  |
| PBGN_5 |  | .79 |  |  |  |  |  |  | .78 |  |  |  |  |  |
| PBGN_6 |  | .78 |  |  |  |  |  |  | .78 |  |  |  |  |  |
| PBGN_7 |  | .76 |  |  |  |  |  |  | .78 |  |  |  |  |  |
| PBGN_8 |  | .76 |  |  |  |  |  |  | .78 |  |  |  |  |  |
| **PBGN_9** |  | .73 |  |  |  |  |  |  | .74 |  |  |  |  |  |
| **PBGN_10** |  | .73 |  |  |  |  |  |  | .73 |  |  |  |  |  |
| **PBGN_11** |  | .67 |  |  |  |  |  |  | .70 |  |  |  |  |  |
| **AF_1** |  |  | .87 |  |  |  |  |  |  | .83 |  |  |  |  |
| **AF_2** |  |  | .87 |  |  |  |  |  |  | .83 |  |  |  |  |
| **AF_3** |  |  | .78 |  |  |  |  |  |  | .79 |  |  |  |  |
| **AF_4** |  |  | .74 |  |  |  |  |  |  | .76 |  |  |  |  |
| **AF_5** |  |  | .72 |  |  |  |  |  |  | .75 |  |  |  |  |
| IC_1 |  |  |  | .94 |  |  |  |  |  |  | .91 |  |  |  |
| **IC_2** |  |  |  | .89 |  |  |  |  |  |  | .89 |  |  |  |
| **IC_3** |  |  |  | .87 |  |  |  |  |  |  | .88 |  |  |  |
| **IC_4** |  |  |  | .77 |  |  |  |  |  |  | .82 |  |  |  |
| **FAE_1** |  |  |  |  | .92 |  |  |  |  |  |  | .86 |  |  |
| **FAE_2** |  |  |  |  | .87 |  |  |  |  |  |  | .84 |  |  |
| **FAE_3** |  |  |  |  | .78 |  |  |  |  |  |  | .80 |  |  |
| **FSS_1** |  |  |  |  |  | .78 |  |  |  |  |  |  | .78 |  |
| **FSS_2** |  |  |  |  |  | .71 |  |  |  |  |  |  | .75 |  |
| **FSS_3** |  |  |  |  |  | .65 |  |  |  |  |  |  | .72 |  |
| **RTGD_1** |  |  |  |  |  |  | .78 |  |  |  |  |  |  | .79 |
| **RTGD_2** |  |  |  |  |  |  | .65 |  |  |  |  |  |  | .76 |
| **RTGD_3** |  |  |  |  |  |  | .62 |  |  |  |  |  |  | .70 |

*Note.* Items in bold were retained in the CFA. ASGSD = School acceptance and support of gender and sexual diversity; PBGN = Popularity based on gender norms; AF = Academic fairness; IC = Inclusive curriculum; FAE = Freedom of appearance expression; FSS = Freedom of subject selection; RTGD = Reinforcement of traditional gender roles. Items in bold were retained in the CFA
