# Supplementary material for: Students’ perceptions of the rules and restrictions of gender at school: A psychometric evaluation of the Gender Climate Scale (GCS)
Source: Front Psychol. 2023 Mar 2;14:1095255. doi: 10.3389/fpsyg.2023.1095255 (PMC10019353; doi:10.3389/fpsyg.2023.1095255)
Supplement: Supplementary file 2 [file Data_Sheet_2.docx]

Appendix B – Final breakdown of the Gender Climate Survey factors and items.

**Factor 1: School acceptance and support of gender and sexual diversity (ASGSD)**

Description: This factor measures how accepting and supportive the school environment is of non-conforming gender expression and diversity, and same sex attraction.

1. ASGSD_2: At my school, gender diverse students are free to change their appearance as they like without being teased.
2. ASGSD_3: At my school, nobody teases students who are gender diverse (e.g. transgender, non-binary).
3. ASGSD_6: Students would feel comfortable to transition their gender at my school.
4. ASGSD_7: In my school, same-sex relationships are respected just as much as opposite-sex relationships.
5. ASGSD_10: At my school, all students are treated equally, regardless of how they express their gender
6. ASGSD_12: At my school, students in same sex relationships are free to hug and kiss one another without being teased.

**Factor 2: Reinforcement of Traditional Gender Difference (RTGD)**

Description: This factor measures the extent to which traditional gender roles are maintained through teacher discourse with students within the school.

1. RTGD_1: My teachers say that men and women are naturally different from one another.
2. RTGD_2: According to my teachers, men and woman are more different than alike.
3. RTGD_3: At my school, teachers say that men and women are each good at different things.

**Factor 3: Freedom of subject selection (FSS)**

Description: This factor assesses whether gender bias relating to subject selection operates within the school.

1. FSS_1: None of the subjects in my school are seen as “boy” or “girl” subjects.
2. FSS_2: People at my school don’t think certain subjects are mostly/just for boys.
3. FSS_3: People at my school don’t think certain subjects are mostly/just for girls.

**Factor 4: Freedom of Appearance Expression (FAE)**

Description: This factor measures the extent that school-based rules and policies prevent students from using their appearance to express their gender.

1. FAE_1: Students at my school can choose the parts of the school uniform (pants or skirt) that they like to wear
2. FAE_2: In my school, I am free to style my hair in any way I might like.
3. FAE_3: In my school, I am free to wear any element of the school uniform (pants or skirt) regardless of my gender.

**Factor 5: Inclusive HPE Curriculum (IC).**

Description: This factor measures the extent to which gender and sexual diversity is incorporated into the school’s Health and Physical Education curriculum.

1. IC_2: I learned about gay male sexuality in my Health and Physical Education class at school.
2. IC_3: I learned about bisexuality in my Health and Physical Education class at school.
3. IC_4: I learned about what it means to be transgender (sometimes shortened to “trans”) in my Health and Physical Education class at school.

**Factor 6: Academic fairness (AF)**

Description: This factor measures students’ perceptions about marks being awarded based on achievement and effort rather than how students express their gender

1. AF_1: Marks in my classes are awarded fairly, regardless of a student’s gender expression.
2. AF_2: In my school, marks are awarded based on effort, not on how a student expresses their gender.
3. AF_3: Student’s gender expression does not affect the marks they get in class.
4. AF_4: Regardless of how students express their gender, all marks in class are given fairly.
5. AF_5: They way students express their gender has nothing to do with the marks they get in class.

**Factor 7: Popularity based on gender norms (PBGN)**

Description: This factor measures the extent to which GSD students perceive popularity in their school to be based on adherence to traditional male and female gender norms.

1. PBGN_1: The most popular girls in my school, are the ones who look like ‘normal’ girls
2. PBGN_2: To be popular at my school, female students need to act just like girls are expected to
3. PBGN_3: To be popular at my school, you need to look like a ‘typical’ girl.
4. At my school, the girls with the most friends are the ones who look and act the most girly
5. PBGN_9: The most popular boys at my school act just like a boy should
6. PBGN_10: To be popular at my school, boys need to look muscular and strong
7. PBGN_11: To be a popular boy at my school, you need to look like a typical boy
